# Cardiometabolic risk reduction through lifestyle intervention programs in the Brazilian public health system

**DOI:** 10.1186/1758-5996-5-21

**Published:** 2013-04-18

**Authors:** Antonela Siqueira-Catania, Adriana Cezaretto, Camila Risso de Barros, Emanuel Péricles Salvador, Tainá Carvalho dos Santos, Sandra RG Ferreira

**Affiliations:** 1 Nutrition Department, School of Public Health, University of São Paulo, Sao Paulo, SP, Brazil

**Keywords:** Lifestyle intervention, Pre-diabetes, Metabolic syndrome, Cardiometabolic risk, Public health system

## Abstract

Public health strategies to reduce cardiovascular morbidity and mortality should focus on global cardiometabolic risk reduction. The efficacy of lifestyle changes to prevent type 2 diabetes have been demonstrated, but low-cost interventions to reduce cardiometabolic risk in Latin-America have been rarely reported. Our group developed 2 programs to promote health of high-risk individuals attending a primary care center in Brazil. This study compared the effects of two 9-month lifestyle interventions, one based on medical consultations (traditional) and another with 13 multi-professional group sessions in addition to the medical consultations (intensive) on cardiometabolic parameters. Adults were eligible if they had pre-diabetes (according to the American Diabetes Association) and/or metabolic syndrome (International Diabetes Federation criteria for Latin-America). Data were expressed as means and standard deviations or percentages and compared between groups or testing visits. A p-value < 0.05 was considered significant. Results: 180 individuals agreed to participate (35.0% men, mean age 54.7 ± 12.3 years, 86.1% overweight or obese). 83 were allocated to the traditional and 97 to the intensive program. Both interventions reduced body mass index, waist circumference and tumor necrosis factor-α. Only intensive program reduced 2-hour plasma glucose and blood pressure and increased adiponectin values, but HDL-cholesterol increased only in the traditional. Also, responses to programs were better in intensive compared to traditional program in terms of blood pressure and adiponectin improvements. No new case of diabetes in intensive but 3 cases and one myocardial infarction in traditional program were detected. Both programs induced metabolic improvement in the short-term, but if better results in the intensive are due to higher awareness about risk and self-motivation deserves further investigation. In conclusion, these low-cost interventions are able to minimize cardiometabolic risk factors involved in the progression to type 2 diabetes and/or cardiovascular disease.

## Introduction

Cardiovascular disease is still a major cause of death [1] and some data have indicated similar impact of type 2 diabetes mellitus (t2DM) on mortality [2]. Public health strategies to reduce cardiovascular morbidity and mortality in populations should focus on global cardiometabolic profile, which include glucose homeostasis.

Lifestyle intervention programs to prevent t2DM have been conducted mainly in developed countries [3,4] but also in developing world [5]. The efficacy of a healthy diet and physical activity has already been consistently demonstrated and the protection conferred is higher than that obtained by pharmacological interventions [4-6]. Cost-effectiveness analyses have also shown the benefits of t2DM prevention for societies [7]. Interestingly, beneficial effects obtained through lifestyle changes are observed even after the interruption of the study protocols [8]. However, it remains a challenge for health professionals and governmental systems to change and sustain healthy habits in populations. Taking these data into consideration, the International Diabetes Federation proposed that each country should develop your own lifestyle intervention program to improve health of individuals at high risk, tailored to local realities [6].

In Latin America, a high prevalence of traditional cardiovascular risk factors such as hypertension, smoking habit and dyslipidemia are still observed, together with the ongoing obesity and t2DM epidemic [9]. Despite this scenario, few preventive programs to reduce cardiometabolic risk in the public health system have been developed, and low-cost interventions are urgently needed. Effective lifestyle modification programs should include a multi-professional team, and in this setting of developing countries, optimizing care through interdisciplinary group sessions would be desirable. Also, a larger approach, aiming at global cardiometabolic risk reduction, would reach a higher number of individuals.

We developed two 9-month lifestyle interventions to promote health of high-risk individuals attending a primary care center in Brazil, feasible to be implemented in the public health system: a traditional program based on medical consultations, and an intensive program, which also included interdisciplinary group sessions with a multi-professional team [10]. Such initiative may represent the first step toward orienting public health programs aimed at improving life habits of Brazilian individuals at cardiometabolic risk.

In addition to classical cardiovascular risk factors, identification of early metabolic disturbances is of great importance in order to direct preventive measures. Once inflammatory cytokines are involved in the genesis of t2DM and atherosclerosis, markers of subclinical inflammation and insulin resistance might be helpful to indicate intervention benefits [11]. The present analysis describes the impact of these intervention programs on several cardiometabolic parameters in high-risk individuals seen by the public health system in Brazil.

## Methods

**Figure 1:**
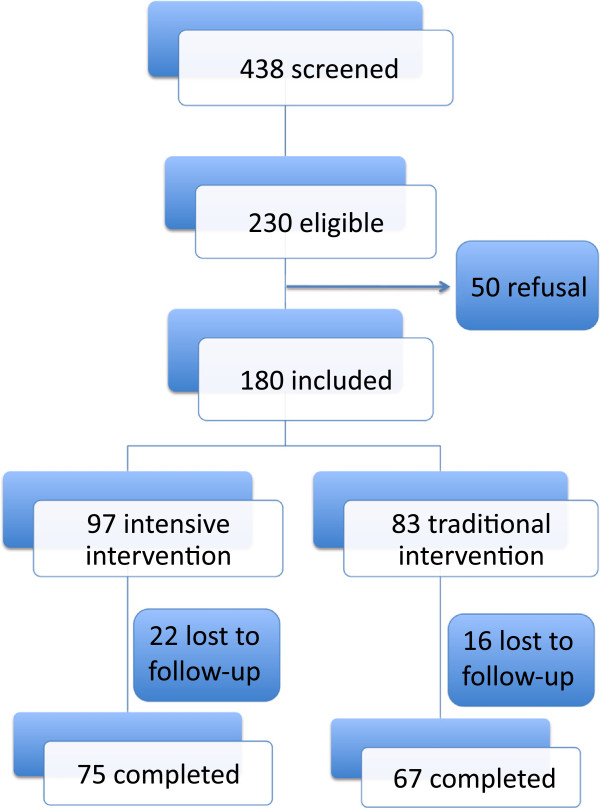
Enrollment and follow-up.

During 2008 and 2009, adults screened by a questionnaire of risk for t2DM were invited to a clinical examination and an oral glucose tolerance test in the School of Public Health care unit of the University of Sao Paulo, Brazil. These individuals, who seek for assistance under the Brazilian public health system, predominantly have low incomes and schooling. Inclusion criteria were age between 18 and 79 years and the presence of prediabetic conditions and/or metabolic syndrome without diabetes. Individuals with a medical history of neurological or psychiatric disturbances, liver, renal or infectious chronic diseases were excluded. For this interventional study, 438 individuals were screened, 230 were eligible, 50 refused to participate and 180 individuals were included in this convenience sample (Figure 1). Among non-participants, there was a predominance of men; however, they did not differ from participants in terms of sociodemographic, anthropometric or metabolic variables. This study was approved by the local Research Ethics Committee and written consent was obtained from all participants.

Trained staff collected physical activity, dietary and clinical data. Body weight was measured in a digital scale balance, height in a manual stadiometer and body mass index (BMI) calculated. Waist circumference was measured with non-distensible tape in the upright position, according to the WHO recommendation [12]. Blood pressure was measured three times, in a sitting position, by automatic device (Omron model HEM-712C, Omron Health Care, Inc, USA). Mean of the two last measures were considered in analyses.

Dietary data were obtained using three 24-hour recalls and energy intake was estimated. Physical activity domains were assessed by the long version of the international physical activity questionnaire [13]. The Medical Outcome Study 36-item Short Form Health Survey (SF-36) was used to assess quality of life, which includes questions focusing on physical and emotional concerns of daily activities (http://www.sf-36.org).

After overnight fasting, participants were submitted to a 75-g oral glucose tolerance test. Fasting blood samples were collected for plasma glucose and lipid profile. Specimens were aliquoted and stored at −80°C for further determinations. Categories of glucose tolerance were defined according to the American Diabetes Association criteria [14]. Individuals with prediabetes (impaired fasting glycemia and/or impaired glucose tolerance) or fulfilling metabolic syndrome criteria according to International Diabetes Federation definition for Latin-American populations [15] were randomly allocated to one of the 9-month lifestyle intervention programs: the traditional or the intensive program. At the end of the follow-up, participants were resubmitted to the same protocol.

In the traditional program, participants received a total of 4 medical consultations with endocrinologist, when counseling for changing living habits was given. In the intensive, they attended the medical consultations plus 13 interdisciplinary group sessions, when topics on healthy diet, physical activity and psychosocial stress management were discussed with a multi-professional team, including a medical doctor, nutritionist, physical educator and psychologist. Every session was conducted with a maximum of 15 participants and had the duration of two hours. Six group sessions occurred during the first two months, when strategies for changing living habits were provided, in addition to practical information. Thereafter, 7 monthly sessions were conducted to reinforce compliance to the skills acquired. Also, telephone calls between sessions aimed at enhancing self-motivation, risk awareness and patient-professional link. Details of the intensive program are described elsewhere [10].

Plasma glucose was measured by the gluco-oxidase method and lipoproteins enzymatically by automatic analyzer. Insulin, C-reactive protein (CRP) and tumor necrosis factor-α (TNF-α) by immunoenzymatic chemoluminescent assay (commercial kits for IMMULITE, Siemens Healthcare Diagnostics Products Ltd, Llamberis, Gwynedd, United Kingdom) and adiponectin by ELISA (Human Adiponectin ELISA Kit, Millipore Corporation, MA, USA). Homeostasis model assessment (HOMA-IR) was calculated to assess insulin resistance [16].

### Statistical analysis

Dietary, physical activity, clinical and laboratorial data were expressed as means and standard deviations or percentages. Dietary data were processed using the Nutrition Data System® software (Minnesota Nutrition Coordinating Center, USA). ANOVA (analysis of variance) was applied to compare mean levels of variables between groups and testing visits (baseline and 9 months of follow-up) and, for those variables that were different in baseline between groups, ANCOVA (analysis of co-variance) was applied to adjust for these differences. Percentages were compared by qui-square. A *p*-value <0.05 was considered significant. Statistics were performed using SPSS, version 17.0 for Windows (SPSS Inc., Chicago, Illinois, USA).

## Results

From 180 individuals who agreed to participate, 83 and 97 were allocated to traditional and intensive program, respectively (Figure 1). The main reason for refusals was a short timeframe to attend the health care unit during business hours. Similar proportions of individuals (22.7% in intensive and 19.2% in the traditional, *p* = 0.432) were lost to follow-up. They did not differ according to age, sex distribution or clinical characteristics from those who completed the intervention program.

At baseline, mean age of participants was 54.7 ± 12.3 years, 35.0% of them were men and 12.8% current smokers. Forty-seven percent of women were post-menopausal. A total of 86.1% were overweight or obese, 61.3% had prediabetes and 89.4% had metabolic syndrome. Individuals allocated to the intensive program had higher frequency of metabolic syndrome, compared to those to the traditional (93.8 *vs.* 84.3%, *p* < 0.05), but they showed comparable frequencies of glucose metabolism disturbances. In baseline, higher mean values of BMI, waist circumference, and diastolic blood pressure were observed in individuals allocated to the intensive program, compared to those to the traditional one (Table 1). They had also higher mean CRP and lower adiponectin concentrations.

**Table 1:** Baseline and 9-month clinical and laboratory data and their changes according to the type of intervention (IP: intensive and TP: traditional)

	**Intensive**			**Traditional**			**p (intensive*vs.*traditional)**
	**Baseline**	**9 months**	**p**	**Baseline**	**9 months**	**p**	
Energy intake (kcal)	1870 ± 77	1519 ± 64	<0.001	1735 ± 83	1539 ± 70	<0.001	0.043
Body mass index (kg/m^2^)	31.7 ± 5.7	31.0 ± 5.7	<0.001	29.9 ± 5.8**	29.4 ± 5.4**	0.002	0.510
Waist circumference (cm)	103.6 ± 11.9	101.0 ± 10.7	<0.001	98.6 ± 13.5**	97.3 ± 12.8**	0.036	0.053
Systolic blood pressure (mmHg)	136.3 ± 17.4	132.2 ± 19.1	0.060	134.3 ± 17.8	134.7 ± 18.9	0.994	0.037
Diastolic blood pressure (mmHg)	84.2 ± 10.0	79.6 ± 8.5	<0.001	80.3 ± 9.9**	79.7 ± 8.1	0.933	<0.001
Fasting plasma glucose (mg/dL)	99.4 ± 12.1	96.7 ± 12.1	0.072	99.1 ± 11.0	98.4 ± 12.8	0.646	0.262
2-h plasma glucose (mg/dL)	121.2 ± 27.6	114.1 ± 28.5	0.047	115.1 ± 26.9	114.2 ± 30.3	0.991	0.088
Fasting insulin (μUI/mL)	10.4 ± 6.9	8.8 ± 6.7	0.089	9.9 ± 6.8	7.8 ± 5.9	0.019	0.569
HOMA-IR	2.55 ± 1.75	2.10 ± 1.61	0.059	2.44 ± 1.79	1.96 ± 1.65	0.059	0.904
Total cholesterol (mg/dL)	201.8 ± 39.8	197.6 ± 39.9	0.258	195.9 ± 44.6	193.6 ± 46.9	0.370	0.748
HDL-cholesterol (mg/dL)	42.7 ± 11.8	45.5 ± 12.6	0.058	41.7 ± 11.8	45.3 ± 12.3	0.011	0.563
Triglycerides (mg/dL)	151.4 ± 66.5	144.7 ± 51.6	0.568	150.4 ± 68.4	152.7 ± 87.8	0.725	0.245
Adiponectin (ng/mL)	11.6 ± 7.2	15.7 ± 9.7	0.018	18.1 ± 17.6**	18.2 ± 17.1	0.999	0.030
TNF-α (ng/mL)	12.6 ± 6.2	11.0 ± 7.2	0.017	12.4 ± 7.2	10.6 ± 5.3	0.004	0.634
C-reactive protein (mg/dL)	0.60 ± 0.56	0.58 ± 0.56	0.963	0.42 ± 0.49**	0.30 ± 0.34**	0.084	0.132

Data expressed as means and standard deviations.

Statistical analysis: repeated measures ANOVA or ANCOVA.

* p < 0.05; **p < 0.001 *vs.* intensive.

BMI: body mass index; WC: waist circumference; TNF: tumor necrosis factor.

In the group sessions of the intensive program, mean rate of participation was 64%. After 9 months, a higher proportion of individuals practiced the minimum of 150 minutes of physical activity per week in intensive program as compared to traditional (25.9 *vs.* 8.3%, *p* < 0.01). Energy intake reduced significantly in both groups of interventions, but response to intensive program was more pronounced (Table 1). Smoking habit reductions were not observed in the intensive (14.4% of smokers at baseline *vs.* 13.3% after 9 months, *p* = 0.896) nor in the traditional program (10.8% of smokers at baseline *vs.* 10.8% after 9 months, *p* = 0.999).

Both interventions induced reductions in BMI, waist circumference and TNF-α concentration (Table 1). However, only in the intensive program, diastolic blood pressure levels, 2-hour plasma glucose and adiponectin concentrations improved significantly (Table 1). HDL-cholesterol concentrations increased only in the traditional program (Table 1).

**Figure 2:**
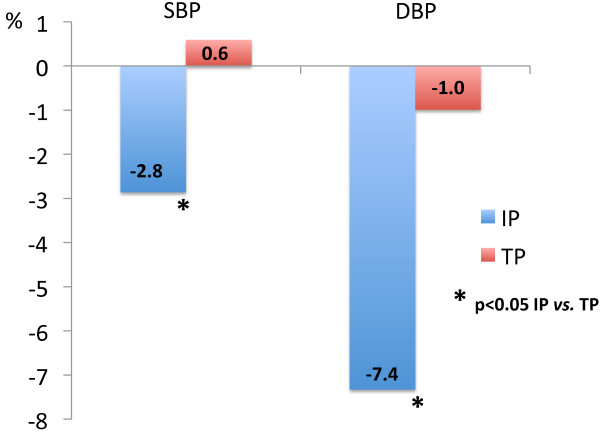
Percent changes in blood pressure (SBP: systolic; DBP: diastolic blood pressure) during 9 months of follow up, according to the intervention program: IP (intensive) and TP (traditional program).

Comparing the responses to the interventions, the intensive program showed better results in terms of blood pressure and adiponectin values (Table 1). The percent improvement in blood pressure levels was significantly higher in the intensive than in the traditional program (Figure 2), as was the increase in adiponectin concentrations (intensive: 51.6 *vs.* traditional: 6.8%, *p* < 0.001). Also, the decrease in plasma glucose levels was higher in intensive, although difference did not reach statistical significance (fasting plasma glucose: 4.0 in intensive *vs.* 1.0% in traditional, *p* = 0.092; 2-hour glucose: 5.8 in intensive *vs.* 2.6% in traditional, *p* = 0.102). Other parameters showed similar changes between groups.

No new case of t2DM was detected in intensive program but three in the traditional during the follow-up and one non-fatal myocardial infarction occurred among individuals from the traditional program.

## Discussion

This real-life prevention study showed that 9-month lifestyle interventions in high-risk adults treated under the public health system of a developing country were able to change their cardiometabolic risk profile. This is of great interest since our low-cost interventions [10] may be useful for Brazilian public health system and for other countries with similar realities. Our findings are in agreement with previous efficacy trials, in which lifestyle modifications were associated with diabetes prevention [2-4]. In comparison to a doctor-centered intervention (traditional program), an interdisciplinary approach considering also psychosocial aspects of the individuals may result in better outcomes in the long term.

In research setting, trials on t2DM prevention conducted in developed world [3,4] and other developing countries [5] proved the efficacy of lifestyle intervention programs. Noteworthy, the Indian experience [5], tailored to middle-class population, showed a lower cumulative incidence of diabetes in the intensive intervention, when compared to the control group, after 3 years of follow-up (55.0% *vs.* 39.3%; p < 0.05). Afterwards, International Diabetes Federation have encouraged the development of t2DM preventive programs, and realistic interventions have been implemented in Europe through the DE-Plan (Diabetes in Europe: Prevention using Lifestyle, Physical Activity and Nutritional intervention Project) and the IMAGE project [17,18].

Trying to meet these IDF recommendations, we developed two modalities of intervention and compared them in the short-term: a traditional, based on medical consultations with endocrinologist, and an intensive, which also had the interdisciplinary group sessions. Both were effective to reduce energy intake, BMI and waist circumference, and even though these were modest changes, a beneficial impact on cardiometabolic risk factors could be observed. Our patients allocated to the intensive program showed a more severe metabolic condition, but apart from worse baseline characteristics, they showed greater benefits compared with the traditional, reinforcing the role of the multiprofessional team and group approach in changing living habits.

Intensive program was more effective than the traditional one to reduce blood pressure levels. These findings were mostly attributed to improvements in compliance with anti-hypertensive medication, possibly as a consequence of higher awareness about their risks. Systemic hypertension is an important cardiometabolic risk factor, so this result has the potential to impact mortality rates in the long term. Analysis of a program implemented in Greek population, with the purpose of preventing t2DM in the clinical setting, also found improvement in systolic blood pressure as a secondary goal [19].

The pathogenetic role of adipocytokines as mediators of obesity-induced insulin resistance and promoters of endothelial dysfunction predisposing individuals to atherosclerosis has been demonstrated [11]. In contrast, adiponectin is shown to improve insulin sensitivity and its concentration is lower in obese than in lean subjects [20]. In our study, even a modest weight loss, induced by changes in diet and physical activity, was capable to improve biomarkers of insulin resistance in the short-term. After 9 months of follow-up, our sample obtained favorable changes in TNF-α and adiponectin concentrations which may have triggered HOMA-IR reductions. Additionally, the intensive program showed to be more effective to promote the desirable elevation in adiponectin. We speculate that these benefits could translate into reductions in t2DM and cardiovascular disease incidence in the long-term.

Similar to our intervention, the GOAL trial implemented a low-cost lifestyle intervention to prevent t2DM [21]. Individuals at high cardiometabolic risk were submitted to 6 group sessions, in which counseling for changing living habits were given by a nurse during a period of 8 months. In contrast, our approach had a higher number of sessions and involved a multiprofessional team, including a psychologist focusing on emotional aspects. In the GOAL trial, the conversion rate to t2DM after 3 years was 12%, an intermediate value between 9% obtained in the intensive intervention and 20% in the control group of the Diabetes Prevention Study [3]. In our study, after 9 months, only three patients from the traditional program progressed to DM and none in the intensive. Although the short duration of our study and sample size did not allow assessing incidence rates, this result may anticipate a protective effect.

The rate of participation on group sessions (64%) was considered satisfactory, provided that patients were adults and sessions were conducted during the business hours. We speculate that the presence of a mental health professional in the team improved compliance, once psychiatric disorders are known to interfere with treatment outcomes [22]. Also, our group has already reported improvements in quality of life in those who participated in the intensive program: role-emotional domain of SF-36 improved in intensive but not in traditional program; and in addition, some positive changes in domains of quality of life correlated to better metabolic outcomes [23]. These results could have impacted self-motivation to adhere with lifestyle changes proposed during the group sessions.

Our study has some limitations. The small sample size and short duration of follow-up did not allow analysis of t2DM or cardiovascular events incidence. Extrapolation to other populations from other developing countries is not possible, but could be tested. At last, it is noteworthy that our reference group was not an ideal control group, considering that medical consultation with an endocrinologist is not commonly available in primary care units. This could have somehow limited the detection of more pronounced differences between modalities of interventions, minimizing the real impact of the intensive intervention in changing living habits.

In summary, both programs, developed to reduce cardiometabolic risk of individuals attending public health system in Brazil, improved metabolic status in the short-term, and the multiprofessional team and group approach induced some better results. We call attention to the potential of these low-cost interventions, feasible in the clinical setting, to promote health of high-risk individuals and maybe slow their progression to t2DM and/or cardiovascular disease.

## Competing interests

The authors declare that they have no conflict of interest.

## Authors’ contributions

SCA carried out the traditional and intensive intervention, participated in data collection, statistical analysis and has written the manuscript. CA carried out the intensive intervention, participated in data collection and helped writting the manuscript. BCR carried out the intensive intervention, participated in data collection and helped writting the manuscript. SEP carried out the intensive intervention and did the statistical analysis. STC carried out the intensive intervention and participated in data collection. FSRG supervised the work and helped writting the manuscript. All authors read and approved the final manuscript.

## Support

FAPESP - Fundação de Apoio à Pesquisa do Estado de São Paulo.
